# Machine learning models using multimodal data accurately predict chemotherapy-induced cardiotoxicity in breast cancer

**DOI:** 10.3389/fcvm.2025.1707889

**Published:** 2026-01-06

**Authors:** Kundi Chen, Yuqiong An, Zhen Wang, Fang Nie

**Affiliations:** Ultrasound Medical Center, Second Hospital of Lanzhou University, Lanzhou, China

**Keywords:** breast cancer, chemotherapy-related cardiac dysfunction, cardiotoxicity, machine learning, prediction model, multimodal data

## Abstract

**Background:**

Despite significant advances in breast cancer therapy, chemotherapy-related cardiac dysfunction (CTRCD) remains a critical clinical challenge. This study aimed to develop and validate machine learning (ML) models that integrate multimodal data to predict the risk of CTRCD in female breast cancer patients.

**Methods:**

We retrospectively analyzed data from 423 female breast cancer patients who received chemotherapy between January 2020 and January 2025. Multimodal data included demographic information, clinical variables, echocardiographic parameters, electrocardiographic (ECG) findings, and cardiac biomarkers. The dataset was randomly split into training and validation sets in a 7:3 ratio. Seven feature selection methods and eight ML algorithms were employed to construct and compare predictive models.

**Results:**

Among the 423 patients, CTRCD occurred in 111 patients (26.24%). Five variables were identified as robust predictors: age, baseline left ventricular ejection fraction <60%, anthracycline–trastuzumab combination therapy, chemotherapy cycles, and abnormal ECG findings. Among all models evaluated, the extreme gradient boosting (XGBoost) algorithm demonstrated the best performance, achieving an area under the curve of 0.782 (95% CI: 0.681–0.883) in 10-fold cross-validation.

**Conclusion:**

The XGBoost-based model showed strong predictive ability and may serve as a practical tool for early risk stratification and timely clinical management of CTRCD.

## 1 Introduction

Breast cancer remains the most prevalent malignancy among women worldwide (1–3). Although chemotherapy has significantly improved survival outcomes, it is frequently associated with cardiotoxic side effects, particularly chemotherapy-related cardiac dysfunction (CTRCD) (4, 5). Among the agents implicated, anthracyclines and trastuzumab are well known for their cardiotoxic potential (6, 7), posing a significant threat to the long-term cardiovascular health of survivors (8).

Early identification of patients at elevated risk for CTRCD is essential to enable timely implementation of preventive and therapeutic strategies, thereby minimizing the likelihood of irreversible cardiac damage (9). However, conventional risk stratification methods—based on clinical parameters, cardiac biomarkers, electrocardiogram (ECG), and echocardiographic assessments—often demonstrate limited predictive accuracy (10–12). These traditional approaches struggle to capture the complex, multifactorial nature of cardiotoxicity, particularly in the context of high-dimensional, heterogeneous data derived from multiple modalities.

In this context, machine learning (ML) has emerged as a promising tool in cardiovascular medicine, capable of processing large-scale multimodal datasets and uncovering subtle, nonlinear patterns that may elude conventional statistical methods (13–15). By integrating clinical, imaging, ECG, and biochemical data, ML-based models offer the potential to improve risk prediction and guide personalized treatment strategies (16).

The present study aimed to develop and validate ML models for predicting CTRCD in breast cancer patients undergoing chemotherapy, using comprehensive multimodal clinical data. To facilitate model interpretability and support clinical translation, we employed SHapley Additive Explanations (SHAP) to assess the relative contribution of each predictor to the output of the final model.

## 2 Materials and methods

### 2.1 Study design and population

This retrospective cohort study was approved by the Ethics Committee of the Second Hospital of Lanzhou University (Approval No. 2023A-435) and conducted in accordance with the Declaration of Helsinki. The requirement for informed consent was waived due to the retrospective design and the use of deidentified data.

We included consecutive female patients diagnosed with breast cancer who received chemotherapy between January 2020 and January 2025. The age of the included patients ranged from 20 to 70 years. Inclusion criteria were as follows: (i) female sex; (ii) no prior history of cardiovascular disease (e.g., congenital heart disease, cardiomyopathy, coronary artery disease, or valvular heart disease); (iii) completion of the entire chemotherapy regimen; (iv) availability of at least two echocardiographic and ECG assessments (baseline and postchemotherapy); and (v) complete clinical and laboratory data.

### 2.2 Data collection

Comprehensive data were extracted from electronic medical records and categorized into five primary domains. Demographic characteristics included age, height, weight, and body surface area. Clinical variables encompassed comorbidities (e.g., hypertension, diabetes, and hyperlipidemia), details of chemotherapy regimens (including drug types, doses, and number of cycles), and histories of radiotherapy and surgery. Echocardiographic parameters focused on baseline left ventricular ejection fraction (LVEF) and its post-treatment changes. ECG findings comprised results from electrocardiograms, noting the presence of arrhythmias or other abnormalities detected during chemotherapy. Lastly, laboratory data included cardiac biomarkers such as high-sensitivity cardiac troponin T (hsTnT) and N-terminal pro-B-type natriuretic peptide (NT-ProBNP), in addition to pathological and immunohistochemical findings.

### 2.3 Endpoint definition

CTRCD was defined according to the evolving consensus of the European Society of Cardiology (ESC) guidelines. The primary criterion, originating from the 2014 ESC Expert Consensus (17) and 2016 ESC Position Paper (18), was a decline in LVEF of ≥10% from baseline to a value <53%, confirmed by repeated echocardiographic assessment. This was supplemented by supportive diagnostic criteria formalized in the 2022 ESC Guidelines on cardio-oncology (9), which include the presence of new or worsening ECG abnormalities (e.g., arrhythmia, ST-T segment changes) and elevated levels of cardiac biomarkers (hsTnT >14 ng/L or NT-proBNP >125 pg/mL). Patients who met the primary LVEF criterion were classified as having CTRCD, irrespective of accompanying ECG or biomarker changes. This ESC guideline-based definition served as the outcome variable for the study.

### 2.4 Feature selection

Feature selection was applied to the structured multimodal dataset. For echocardiographic data, the inputs were quantitatively derived parameters (e.g., baseline LVEF value) rather than features extracted directly from raw imaging data. Seven feature selection methods, representing four major categories, were employed to identify the most relevant predictors: least absolute shrinkage and selection operator (LASSO) regression; three variants of stepwise regression (SR): forward selection (SR-FS), backward selection (SR-BS), and bidirectional elimination (SR-BE); two implementations of recursive feature elimination (RFE): support vector machine-RFE (SVM-RFE) and random forest-RFE (RF-RFE); and optimal subset regression (OSR).

From these methods, nine candidate predictors were identified. Among these, five variables were consistently selected across all methods and were subsequently used for model development.

### 2.5 Model development and evaluation

Model performance was comprehensively evaluated across three key dimensions. Discrimination was assessed using the area under the receiver operating characteristic (ROC) curve (AUC), sensitivity, specificity, positive predictive value, negative predictive value, F1 score, and the optimal cutoff value, with AUC comparisons conducted using the DeLong test. Calibration was evaluated through calibration plots and Brier scores. Clinical utility was assessed via decision curve analysis (DCA) to quantify the net clinical benefit across a range of threshold probabilities.

To enhance model interpretability, SHAP were applied to the best-performing model. SHAP values were utilized for two primary purposes: first, to rank features globally based on their average absolute SHAP value and, second, to explore individual predictions through SHAP waterfall and force plots.

**Figure 1:**
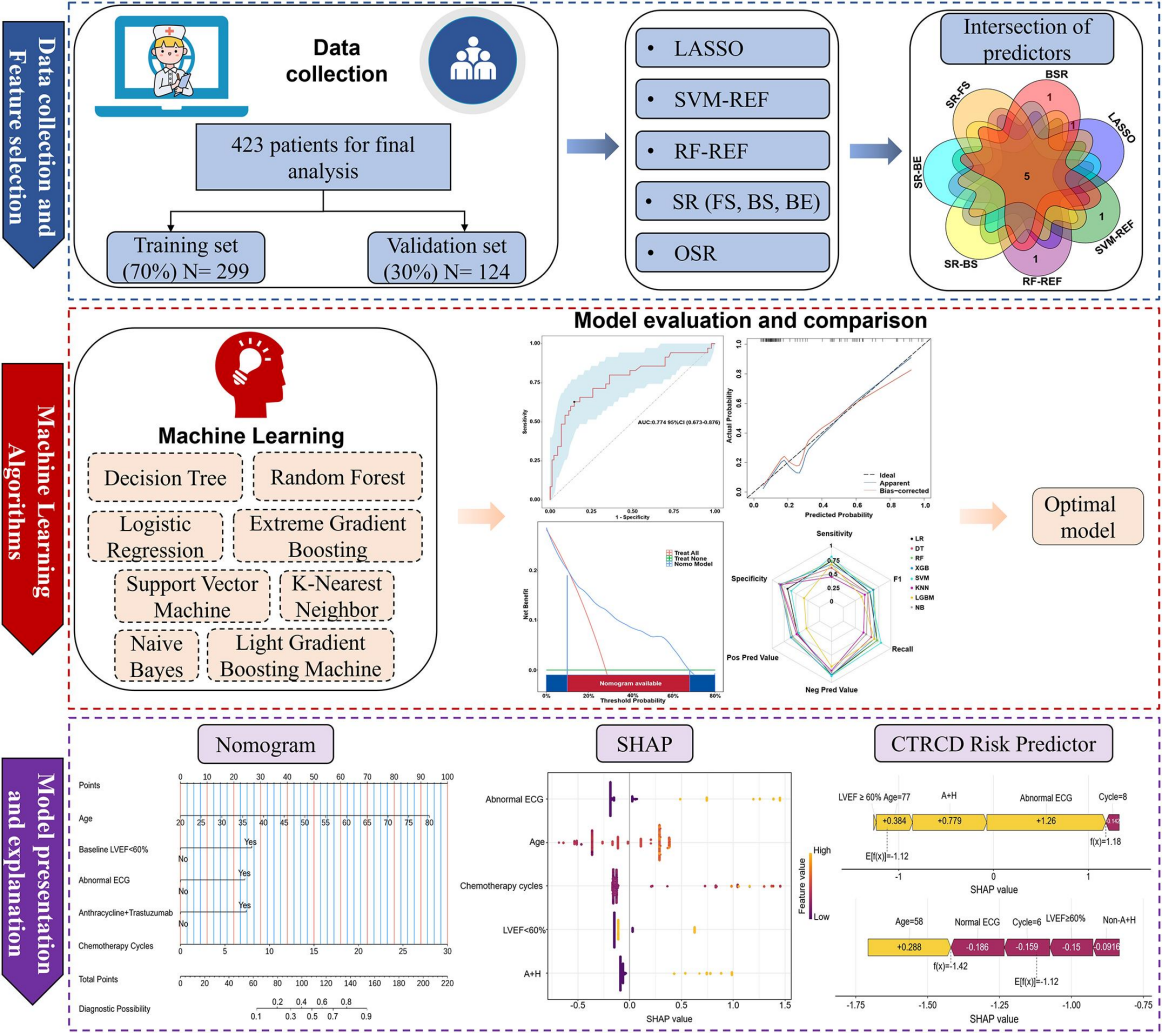
Flowchart of data collection, processing, and analysis. LASSO, least absolute shrinkage and selection operator; SVM-REF, support vector machine–recursive feature elimination; RF-REF, random forest–recursive feature elimination; SR, stepwise regression; FS, forward selection; BS, backward selection; BE, bidirectional elimination; OSR, optimal subset regression; LVEF, left ventricular ejection fraction; ECG, electrocardiogram; A + H, Anthracyclines and Trastuzumab; SHAP, shapley additive explanations; CTRCD, chemotherapy-related cardiac dysfunction.

An overview of the analytical workflow is provided in Figure 1.

### 2.6 Statistical analysis

All statistical analyses were conducted using R software (version 4.2.1) and DCPM (version 4.01; Jingding Medical Technology Co., Ltd.). During preprocessing, variables with >10% missing values or identified outliers were excluded. For those with <10% missingness, missing values were imputed using multiple imputation.

Normality of continuous variables was assessed using the Shapiro–Wilk test. Normally distributed variables were presented as mean ± standard deviation, and compared using two-tailed *t*-tests. Non-normally distributed variables were expressed as median (interquartile range) and compared using the Kruskal–Wallis test. Categorical variables were summarized as frequencies and percentages, and compared using the chi-square test.

Univariate and multivariate logistic regression analyses were performed to identify independent predictors of CTRCD. Multicollinearity was assessed using variance inflation factor and tolerance statistics. A two-sided *P*-value <0.05 was considered statistically significant.

## 3 Results

### 3.1 Baseline characteristics analysis

Of the 768 women diagnosed with breast cancer who received chemotherapy between January 2020 and January 2025 (a 5-year period), 345 were excluded due to incomplete data. In particular, 75 patients received treatment at other hospitals, 73 did not complete the full course of chemotherapy, 60 declined chemotherapy, 58 had inadequate echocardiographic data, 41 lacked cardiac biomarker results, and 38 had incomplete ECG records. Consequently, 423 patients were included in the final analysis.

The baseline characteristics of these patients, stratified into training and validation sets, are summarized in Table 1. The two sets were well balanced, with no statistically significant differences observed across any baseline variable (all *P* > 0.05).

**Table 1:** Baseline characteristics of breast cancer patients who underwent chemotherapy.

Characteristics	All patients, *N* = 423	Training set, *N* = 299	Validation set, *N* = 124	*P-*value
Age, years	50.90 ± 9.54	50.41 ± 9.49	52.06 ± 9.62	0.108
BSA, m^2^	1.63 (1.55, 1.71)	1.63 (1.55, 1.70)	1.62 (1.54, 1.73)	0.675
Hypertension, *n* (%)	36 (8.51)	23 (7.69)	13 (10.48	0.456
Diabetes, *n* (%)	15 (3.55)	10 (3.34)	5 (4.03)	0.775
Hyperlipidemia, *n* (%)	12 (2.84)	8 (2.68)	4 (3.23)	0.753
Estrogen receptor, *n* (%)				0.728
Negative	133 (31.44)	92 (30.77)	41 (33.06)	
Positive	290 (68.56)	207 (69.23)	83 (66.94)	
Progesterone receptor, *n* (%)				0.181
Negative	226 (53.43)	153 (51.17)	73 (58.87)	
Positive	197 (46.57)	146 (48.83)	51 (41.13)	
HER2, *n* (%)				0.694
Negative	151 (35.70)	109 (36.45)	42 (33.87%)	
Positive	272 (64.30)	190 (63.55)	82 (66.13%)	
Baseline LVEF, *n* (%)				0.734
≥60%	347 (82.03)	247 (82.61)	100 (80.65)	
<60%	76 (17.97)	52 (17.39)	24 (19.35)	
Cumulative anthracycline dose, mg/m^2^	136.36 (0, 249.69)	134.94 (0, 49.26)	146.54 (0, 251.32)	0.327
Cumulative trastuzumab dose, mg	0 (0, 675)	0 (0,416)	0 (0, 1,203)	0.766
Anthracycline and trastuzumab combination therapy, *n* (%)	34 (8.04)	24 (8.03)	10 (8.06)	1.000
Cycles of chemotherapy, *n* (%)	6 (6, 8)	6 (6, 8)	6 (6, 8)	0.594
Surgery, *n* (%)	399 (94.33)	283 (94.65)	116 (93.55)	0.830
Radiation therapy, *n* (%)	174 (41.13)	122 (40.80)	52 (41.94)	0.915
Electrocardiography, *n* (%)				0.670
Normal	369 (87.23)	259 (86.62)	110 (88.71)	
Abnormal	54 (12.77)	40 (13.38)	14 (11.29)	
hsTnT, *n* (%)				0.166
Normal	413 (97.64)	294 (98.33)	119 (95.97)	
Abnormal	10 (2.36)	5 (1.67)	5 (4.03)	
NT-ProBNP, *n* (%)				0.234
Normal	403 (95.27)	282 (94.31)	121 (97.58)	
Abnormal	20 (4.73)	17 (5.69)	3 (2.42)	

BSA, body surface area; HER2, human epidermal growth factor receptor 2; LVEF, left ventricular ejection fraction; hsTnT, high-sensitivity cardiac troponin T; NT-ProBNP, N-terminal pro-B-type natriuretic peptide.

Of the 423 patients, 111 (26.24%) developed CTRCD. Within this group, 54 patients (48.64%) presented with arrhythmias, including nodal tachycardia, bradycardia, atrioventricular block, premature beats, atrial fibrillation, or ventricular fibrillation; 35 patients (31.53%) met the primary echocardiographic criterion for CTRCD (a decline in LVEF of ≥10% to a value <53%); and 22 (19.82%) developed clinical heart failure classified as New York Heart Association class III or IV.

Regarding treatment modalities among the 423 included patients, 399 (94.33%) received breast radiotherapy, while 174 (41.13%) underwent surgical intervention. Many patients received both treatments as part of their comprehensive cancer care. As for cardiovascular risk factors, 36 patients (8.51%) had hypertension, 15 (3.55%) had diabetes, and 12 (2.84%) had hyperlipidemia.

Table 2 presents the results of univariate and multivariate logistic regression analyses performed in breast cancer patients undergoing chemotherapy. Both analyses identified several variables significantly associated with the development of CTRCD. These variables included age (OR = 1.078, 95% CI: 1.041–1.118, *P* < 0.001), baseline LVEF < 60% (OR = 3.636, 95% CI: 1.754–7.579, *P* < 0.001), anthracyclines–trastuzumab combination therapy (OR = 3.216, 95% CI: 1.159–8.762, *P* = 0.022), number of chemotherapy cycles (OR = 1.175, 95% CI: 1.080–1.302, *P* = 0.001), and abnormal ECG findings (OR = 3.289, 95% CI: 1.474–7.396, *P* = 0.004).

**Table 2:** Univariate and multivariate regression analyses of breast cancer patients who underwent chemotherapy.

Characteristics	Univariate regression		Multivariate regression	
	OR (95% CI)	*P*-value	OR (95% CI)	*P*-value
Age	1.06 (1.03–1.093)	<0.001	1.078 (1.041–1.118)	<0.001
BSA	0.169 (0.016–1.704)	0.136		
Hypertension	1.312 (0.487–3.214)	0.566		
Diabetes	1.268 (0.268–4.691)	0.736		
Hyperlipidemia	0.977 (0.141–4.349)	0.978		
Estrogen receptor	1.032 (0.591–1.841)	0.912		
Progesterone receptor	1.065 (0.632–1.795)	0.813		
HER2	0.907 (0.532–1.563)	0.721		
Baseline LVEF < 60%	3.94 (2.107–7.402)	<0.001	3.636 (1.754–7.579)	0.001
Cumulative anthracycline dose	1 (0.999–1.002)	0.658		
Combine anthracycline and trastuzumab therapy	3.297 (1.401–7.771)	0.006	3.216 (1.159–8.762)	0.022
Cycles of chemotherapy	1.16 (1.08–1.262)	<0.001	1.175 (1.080–1.302)	0.001
Surgery	0.737 (0.258–2.404)	0.583		
Radiation therapy	1.242 (0.732–2.1)	0.419		
Abnormal electrocardiogram	5.259 (2.638–10.69)	<0.001	3.289 (1.474–7.396)	0.004

BSA, body surface area; HER2, human epidermal growth factor receptor 2; LVEF, left ventricular ejection fraction.

**Figure 2:**
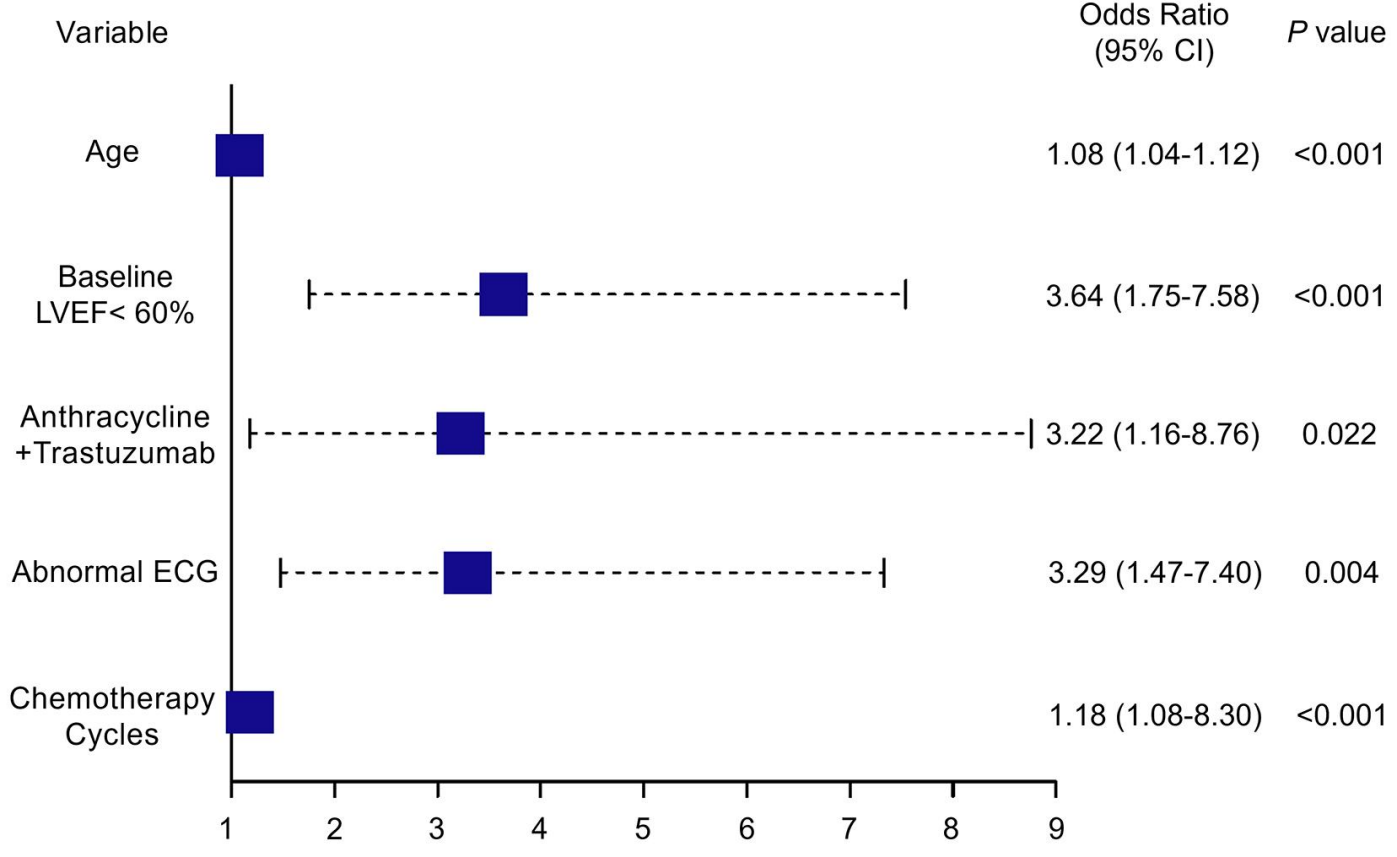
Forest plot depicting independent risk factors for CTRCD identified through multivariate analysis. LVEF, left ventricular ejection fraction; ECG, electrocardiogram; CTRCD, chemotherapy-related cardiac dysfunction; CI, confidence interval. Values in parentheses represent the 95% CI.

These findings indicate that both clinical and treatment-related factors significantly contribute to the risk of CTRCD. The odds ratios derived from the logistic regression model are visualized in the forest plot (Figure 2).

### 3.2 Feature selection

**Figure 3:**
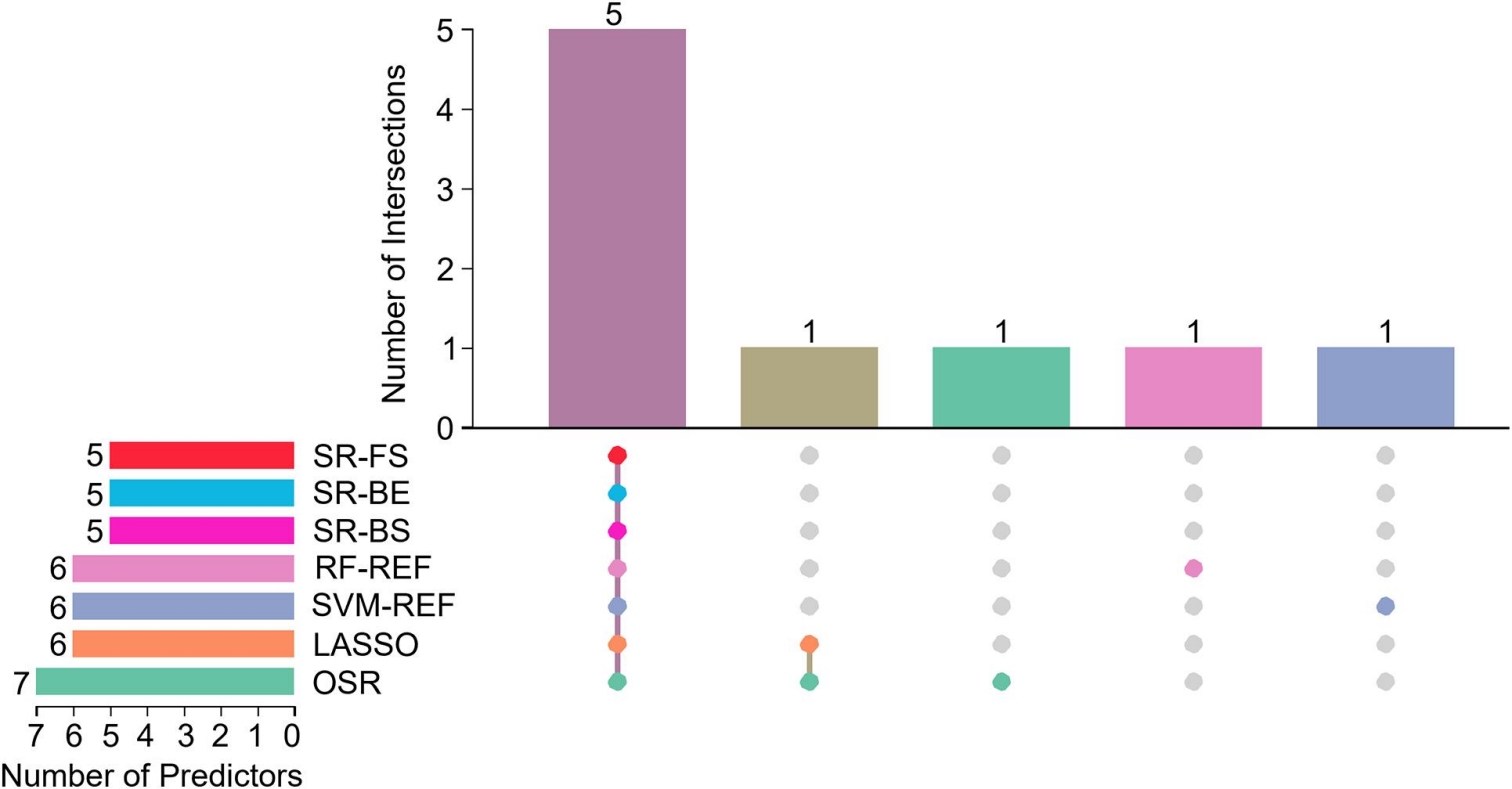
Upset plot of interactions between the predictors. SR, stepwise regression; FS, forward selection; BS, backward selection; BE, bidirectional elimination; RF-REF, random forest–recursive feature elimination; SVM-REF, support vector machine–recursive feature elimination; LASSO, least absolute shrinkage and selection operator; OSR, optimal subset regression.

**Figure 4:**
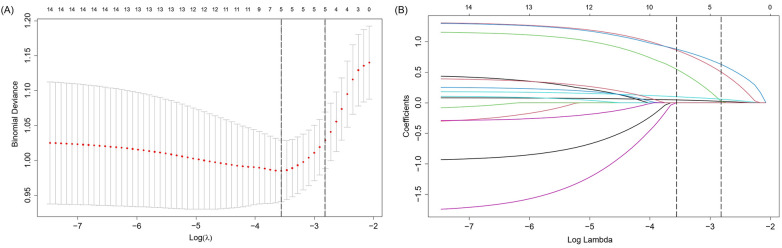
Presentation of the results of the LASSO regression analysis. **(A)** LASSO regression model factor selection: Left dashed line represents the optimal lambda value (lambda⋅ min), while the right dashed line marks the lambda value within one standard error of the optimal (lambda.1se); **(B)** LASSO regression model screening variable trajectories. LASSO, least absolute shrinkage and selection operator.

Figure 3 depicts the overlap of predictors selected by seven different feature selection methods: SR-FS, SR-BS, SR-BE, LASSO, SVM-RFE, RF-RFE, and OSR. The number of selected predictors varied slightly across methods: Five predictors were selected by SR-FS, SR-BS, and SR-BE; six predictors by LASSO (as depicted in Figure 4), SVM-RFE, and RF-RFE; and seven predictors by OSR.

To improve model stability and mitigate selection bias, we identified the intersection of predictors consistently selected across all seven methods. After accounting for overlaps among the variables selected by different methods, the seven feature selection methods collectively identified a consolidated pool of nine unique candidate predictors. Ultimately, five predictors met this criterion and were incorporated into the final model: age, baseline LVEF, abnormal ECG findings, anthracyclines–trastuzumab combination therapy, and number of chemotherapy cycles. These variables were considered the most reliable and clinically relevant features of CTRCD. Importantly, they represent baseline risk factors used to predict the occurrence of the CTRCD endpoint, which was defined separately.

### 3.3 Model development and comparison

Eight ML algorithms were trained on the dataset of 423 patients, split into a training set (70%) and validation set (30%): LR, DT, RF, XGBoost, SVM, KNN, NB, and LGBM. Model performance on the validation set is summarized in Table 3. Among these models, XGBoost and SVM algorithms demonstrated superior sensitivity, balanced accuracy, and F1 scores:

- *XGBoost*: sensitivity = 0.657, balanced accuracy = 0.744, F1 score = 0.630;
- *SVM*: sensitivity = 0.800, balanced accuracy = 0.698, F1 score = 0.566.

**Table 3:** Comparison of performance metrics among eight machine learning models.

Model	AUC	Sensitivity	Specificity	PPV	NPV	F1 score	Brier score	Balanced accuracy
XGBoost	0.782	0.657	0.831	0.605	0.86	0.63	0.156	0.744
SVM	0.778	0.8	0.596	0.438	0.883	0.566	0.166	0.698
LR	0.77	0.714	0.674	0.463	0.857	0.562	0.161	0.694
RF	0.751	0.714	0.798	0.581	0.877	0.641	0.177	0.756
KNN	0.728	0.429	0.82	0.484	0.785	0.455	0.224	0.624
NB	0.674	0.486	0.854	0.567	0.809	0.523	0.214	0.67
DT	0.627	0.593	0.804	0.457	0.876	0.516	0.185	0.698
LGBM	0.506	0.657	0.326	0.277	0.707	0.39	0.213	0.491

AUC, area under the curve; PPV, positive predictive value; NPV, negative predictive value; XGBoost, extreme gradient boosting; SVM, support vector machine; LR, logistic regression; RF, random forest; KNN, K-nearest neighbor; NB, naive bayes; DT, decision tree; LGBM, light gradient boosting machine.

**Figure 5:**
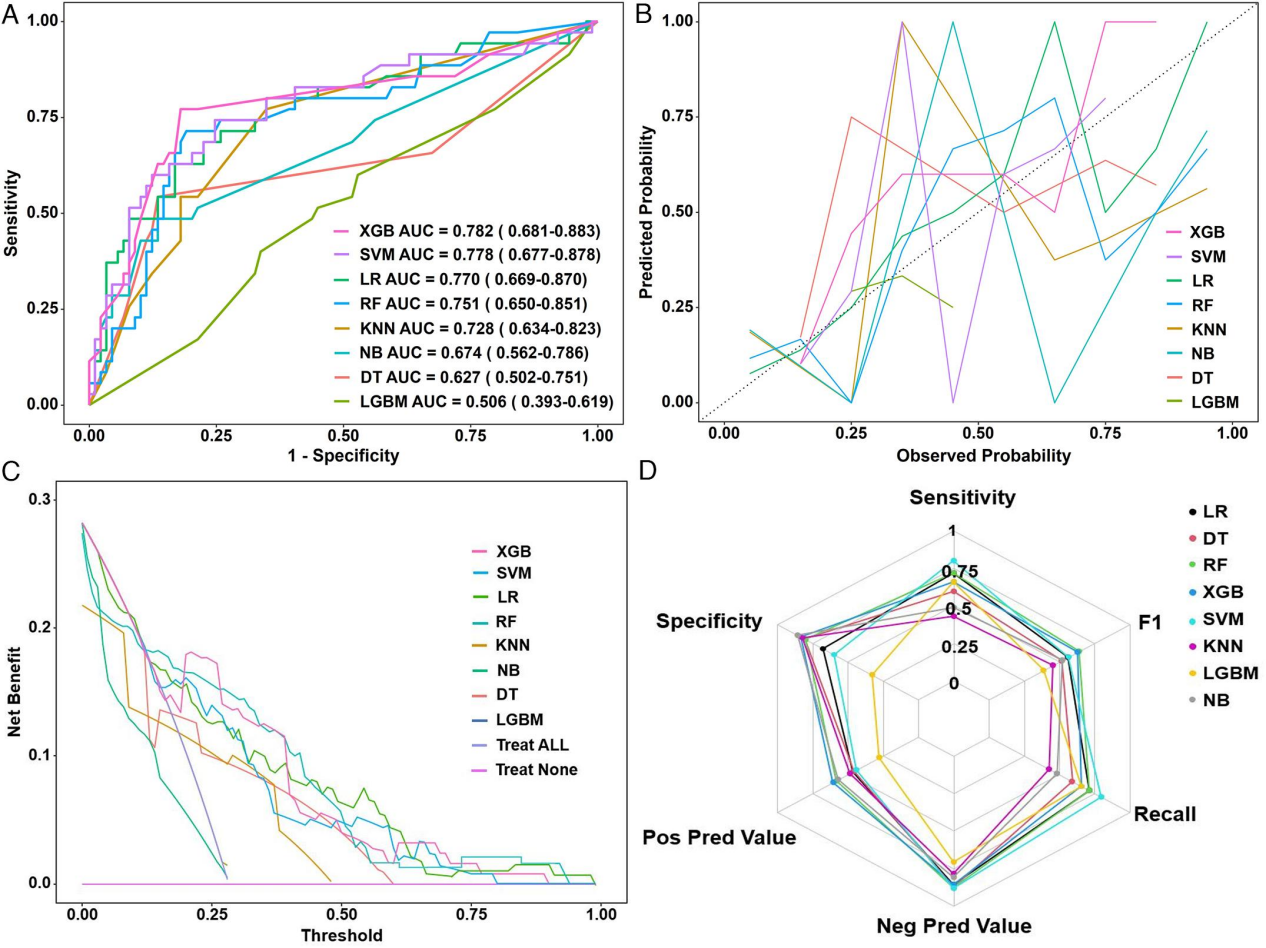
**(A)** ROC curves, **(B)** calibration plots, **(C)** decision curve analysis, and **(D)** radar chart of eight machine learning models in the validation set. ROC, receiver operating characteristic curve; XGB, extreme gradient boosting; SVM, support vector machine; LR, logistic regression; RF, random forest; KNN, K-nearest neighbor; NB, naive Bayes; DT, decision tree; LGBM, light gradient boosting machine; AUC, area under the receiver operating curve.

ROC analysis revealed that the AUC ranged from 0.506 to 0.782 (Figure 5A). The DeLong test indicated that XGBoost (AUC = 0.782; 95% CI: 0.681–0.883) and SVM (AUC = 0.778; 95% CI: 0.677–0.878) significantly outperformed other models (LR = 0.770, RF = 0.751, KNN = 0.725, NB = 0.674, DT = 0.627, LGBM = 0.506; *P* < 0.05; Table 3).

Calibration curves demonstrated good agreement between predicted and observed outcomes, with XGBoost achieving the lowest Brier score (0.156; 95% CI: 0.122–0.193), indicating excellent model calibration (Figure 5B). DCA further underscored the clinical utility of XGBoost, which exhibited the greatest net benefit across a range of threshold probabilities (Figure 5C). According to the comprehensive performance assessment illustrated by the radar plot (Figure 5D), the XGBoost algorithm outperformed the other models, exhibiting superior sensitivity, specificity, positive predictive value (PPV), negative predictive value (NPV), recall, and F1 score.

Collectively, these results suggest that XGBoost offers the most balanced performance profile and may be the optimal choice for CTRCD prediction in breast cancer patients receiving chemotherapy. However, the optimal model may vary depending on specific clinical goals, such as prioritizing sensitivity or specificity.

### 3.4 Optimal model interpretation

**Figure 6:**
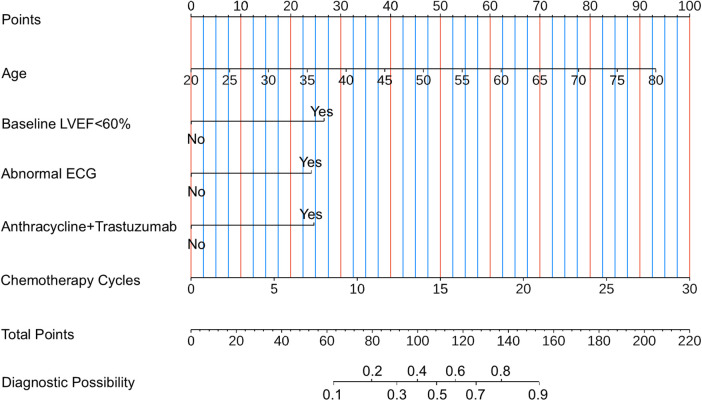
Nomogram for estimating the risk of CTRCD. To use the nomogram, follow these three steps: (i) Identify the patient's value for each predictor on its corresponding axis and draw a vertical line upward to the “Points” axis to determine the score assigned to that variable. (ii) Sum the individual scores to obtain the “Total Points.” (iii) Locate the total score on the “Total Points” axis and draw a vertical line downward to estimate the corresponding probability of CTRCD. LVEF, left ventricular ejection fraction; ECG, electrocardiogram; CTRCD, chemotherapy-related cardiac dysfunction.

To facilitate convenient and individualized risk assessment of CTRCD, we developed two types of nomograms. Both static and interactive nomograms were constructed based on coefficients derived from the logistic regression model. The static nomograms provide a visual reference for estimating CTRCD risk using fixed point-based scoring systems (Figure 6). In contrast, the interactive nomograms (https://ayq-2025.shinyapps.io/CTRCD_Project/) allow users to input patient-specific values for key predictors (e.g., age, baseline LVEF <60%, anthracyclines–trastuzumab combination therapy, chemotherapy cycles, and abnormal ECG) to obtain real-time, individualized risk predictions.

**Figure 7:**
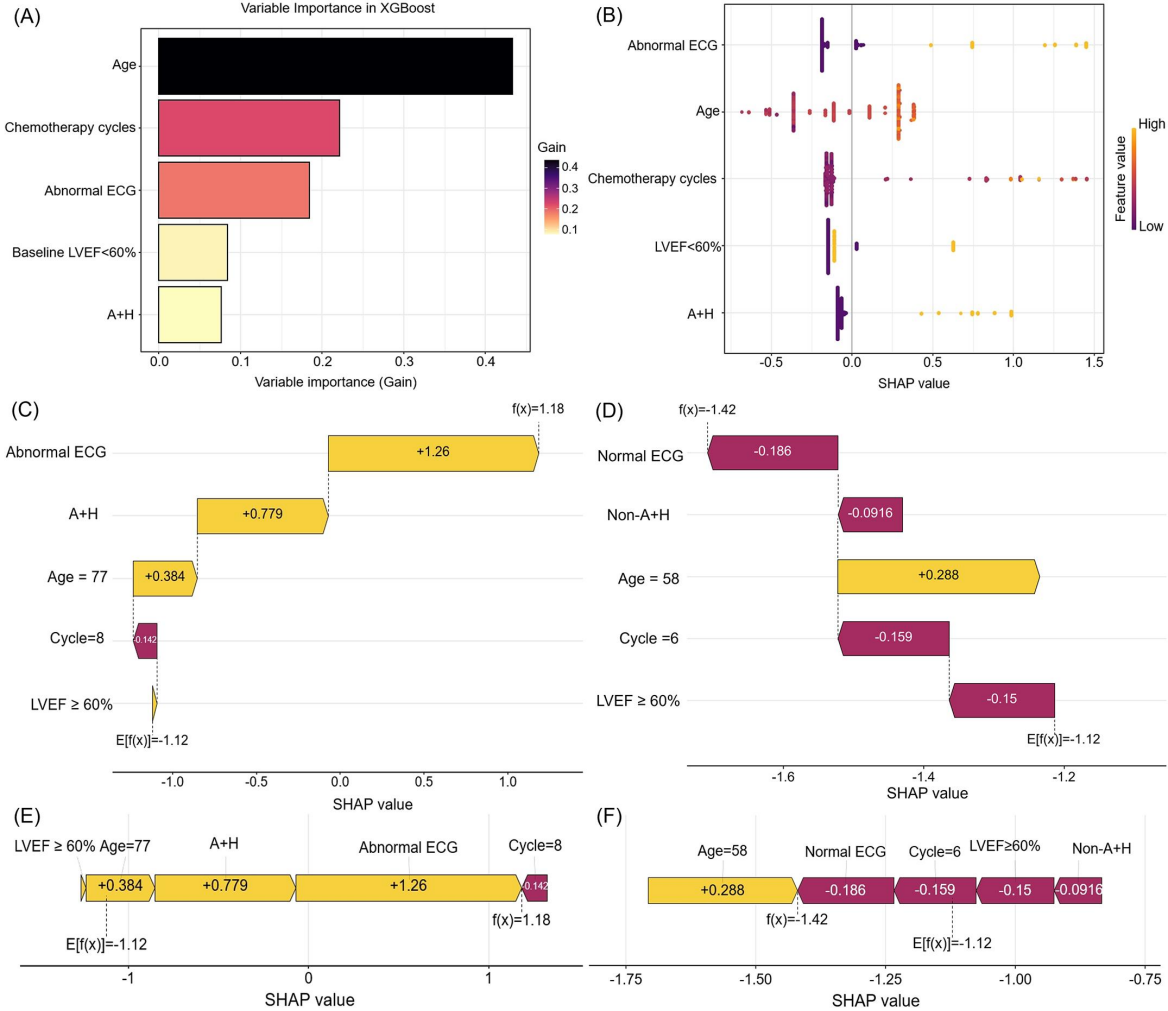
The feature importance and interpretation of the model (based on the XGBoost algorithm). **(A)** The importance ranking of features based on the SHAP value. **(B)** A summary plot of the SHAP values for each feature. **(C,E)** SHAP force plot and waterfall plot for a woman who developed CTRCD. **(D,F)** SHAP force plot and waterfall plot for a woman without CTRCD. SHAP, shapley additive explanations. ECG, electrocardiogram. LVEF, left ventricular ejection fraction. A + H, Anthracyclines and Trastuzumab. CTRCD, chemotherapy-related cardiac dysfunction.

For interpretability of the XGBoost model, SHAP values were employed. The SHAP feature importance plot (Figure 7A) and SHAP summary plot (Figure 7B) ranked the five most influential predictors: age, chemotherapy cycles, abnormal ECG, baseline LVEF <60%, and anthracyclines–trastuzumab combination therapy.

The SHAP waterfall plot (Figure 7C) visualizes SHAP values for a representative patient who developed CTRCD. Key contributors—including abnormal ECG, anthracycline–trastuzumab combination therapy, advanced age (77 years), and preserved baseline LVEF—collectively increased the SHAP value by 1.18, leading to a correct CTRCD prediction. Conversely, the SHAP waterfall plot (Figure 7D) depicts a correctly predicted non-CTRCD case, where normal ECG, fewer chemotherapy cycles, and preserved LVEF contributed to a SHAP value decrease of −1.42.

SHAP force plots (Figures 7E,F) further elucidate individual feature contributions. Yellow bars denote risk factors, purple bars indicate protective factors, and bar length reflects the magnitude of each feature's influence. These visualizations enhance the model's clinical interpretability and provide transparent, patient-specific risk profiling.

## 4 Discussion

In this study, we developed and validated eight ML models to predict CTRCD in female patients with breast cancer, using a comprehensive multimodal dataset that included clinical features, echocardiography, ECG, and biomarkers. Our findings demonstrate that the XGBoost model achieved the best overall performance, achieving an AUC of 0.782, balanced accuracy of 0.744, and the lowest Brier score in the validation set, indicating both strong discriminative power and excellent calibration. These results suggest that ML models, particularly XGBoost algorithm, can be effectively leveraged for individualized CTRCD risk prediction and may support timely clinical decision-making.

Our feature selection process identified five key predictors: age, baseline LVEF <60%, abnormal ECG findings, anthracyclines–trastuzumab combination therapy, and the number of chemotherapy cycles. These variables were consistently selected across all seven feature selection methods, underscoring their statistical robustness and clinical relevance. Notably, baseline LVEF <60% exhibited the strongest association with CTRCD (OR = 3.636, *P* < 0.001) in multivariate logistic regression, reaffirming the critical role of pretreatment echocardiographic assessment in identifying patients at elevated risk (5). Since CTRCD often evolves subclinically, baseline and serial echocardiographic evaluations are essential for the timely detection of cardiac dysfunction and initiation of preventive measures (19).

Furthermore, our identification of anthracycline–trastuzumab combination therapy and increased chemotherapy cycles as independent risk factors aligns with prior studies highlighting the cumulative and dose-dependent cardiotoxicity of these agents (20–22). Our model refines this understanding by quantifying these risks within a contemporary, real-world cohort and integrating them into an individualized risk stratification framework. By integrating therapeutic, clinical, and imaging variables into a unified model, we offer a more comprehensive and individualized risk stratification approach that aligns with the evolving paradigm of precision cardio-oncology.

In comparing models, we observed that while the SVM model achieved the highest sensitivity (0.800), it had lower specificity (0.596) and precision (0.438), resulting in a relatively low F1 score (0.566) and limited clinical utility, as evidenced by decision curve analysis. In contrast, the XGBoost model demonstrated a more balanced performance, with a sensitivity of 0.657 and a high specificity of 0.831. This balance led to a superior PPV (0.605), an improved F1 score (0.630), and the lowest Brier score (0.156) among all models, indicating excellent calibration and overall reliability. These results highlight the value of ensemble learning algorithms such as XGBoost, which are well suited to high-dimensional, heterogeneous clinical datasets due to their capacity to model complex non-linear relationships and interactions (23, 24). The SVM model achieved the highest sensitivity, which is a potential advantage in clinical contexts where missing a CTRCD case is unacceptable. However, the XGBoost model demonstrated a more balanced performance with higher specificity, thus reducing the need for unnecessary cardiac evaluations or treatment modifications. Thus, the choice of predictive model should be strategic and guided by specific clinical objectives and risk tolerance.

Importantly, a notable strength of this study is the integration of SHAP values, which significantly enhance the transparency and interpretability of ML models. While ML is often criticized for being a “black box” (25), SHAP provides an intuitive, quantitative framework that decomposes model predictions into individual feature contributions (26, 27). This enables clinicians to understand why a patient is classified as high or low risk, facilitating greater trust and real-world applicability (28, 29).

From a clinical perspective, patient-specific explanations generated by SHAP have several practical implications. First, they enable identification of modifiable or avoidable risk factors (30, 31), such as reducing chemotherapy cycles or opting for less cardiotoxic regimens in patients near a decision threshold. Second, they support shared decision-making by visually communicating personalized risks through tools like SHAP waterfall plots and force plots (32–34). For instance, our model correctly identified age, abnormal ECG findings, and anthracycline–trastuzumab combination therapy as major contributors to high CTRCD risk in a representative high-risk patient, thereby guiding targeted cardiac surveillance or prophylaxis.

From a research standpoint, SHAP values also offer deeper insights beyond traditional metrics. They reveal non-linear patterns, threshold effects, and complex feature interactions that are not captured by traditional models (35, 36). For example, SHAP can uncover unexpected associations or hidden interactions, generating new hypotheses for mechanistic studies or therapeutic optimization (37, 38). Importantly, SHAP also aids in model validation and auditing by highlighting mismatches between predicted and observed outcomes. In cases of prediction error, SHAP visualizations can help discern whether discrepancies stem from missing data, model bias, or outliers, thus supporting iterative refinement and enhancing model robustness (39).

In summary, the use of SHAP not only boosts the transparency and interpretability of the XGBoost model but also bridges the translational gap between ML and evidence-based medicine (40, 41). Its incorporation makes the predictive tool not only accurate but also actionable, auditable, and clinically meaningful—key attributes for integration into real-world cardio-oncology workflows.

In addition, our study offers several novel contributions to the existing literature. First, we systematically applied and compared multiple feature selection techniques, which increased the model robustness and ensured reproducibility. Second, we integrated multimodal input data—including clinical variables, echocardiographic findings, ECG, and biomarkers—whereas prior studies have primarily focused on single data domains such as echocardiographic parameters or biomarkers alone (42–44). Third, the implementation of a dynamic nomogram and a web-based prediction tool (https://ayq-2025.shinyapps.io/CTRCD_Project/) further enhances the clinical utility of our model. By allowing clinicians to input individual patient parameters and receive real-time CTRCD risk estimates, these tools can facilitate point-of-care decision-making. This individualized approach may guide adjustments in chemotherapy regimens, prompt initiation of cardioprotective agents, or determine the frequency of cardiac surveillance, ultimately improving outcomes in high-risk patients. For example, a 65-year-old patient with abnormal ECG findings and a baseline LVEF <60% was estimated to have a 35.3% probability of developing CTRCD, underscoring the necessity of early intervention and closer cardiac surveillance.

This endeavor aligns with several ongoing research efforts aimed at identifying novel biomarkers for CTRCD. For instance, studies are exploring the utility of multi-omics approaches, high-sensitivity troponin and natriuretic peptide kinetics, and the application of artificial intelligence to standard electrocardiograms for the early detection of subclinical cardiac dysfunction (12, 44). The integration of such promising biomarkers from ongoing studies into future iterations of our model holds significant potential.

Our findings have important implications for clinical practice. Early identification of high-risk patients using the XGBoost model can inform timely interventions, such as intensified cardiac monitoring (e.g., serial echocardiography or biomarker surveillance), initiation of cardioprotective therapies, or adjustment of chemotherapy regimens to reduce cardiotoxicity. Given that CTRCD frequently develops insidiously and remains subclinical in its early stages, predictive models like ours have the potential to enable preemptive clinical action, thereby reducing the likelihood of progression to symptomatic heart failure.

Despite these strengths, several limitations warrant consideration. First, our study was conducted at a single center with a moderate sample size (*n* = 423), which may limit the generalizability of the findings. Future multicenter external validation studies with larger and more diverse populations, as well as prospective validation trials, are essential to confirm the model's performance and clinical utility before widespread adoption. Second, although our dataset included key clinical and imaging features, certain advanced echocardiographic markers (such as GLS) were not routinely available in our retrospective cohort. Incorporating such quantitative imaging parameters in future studies may further enhance model performance and allow for earlier prediction of CTRCD. Furthermore, the predictive model was developed using a predefined set of clinical, echocardiographic, and ECG variables, which may not fully capture the multifactorial nature of CTRCD. Its performance was evaluated against a CTRCD definition centered on LVEF decline. Future prospective studies should therefore aim to discover and integrate novel predictors (e.g., GLS) and validate the model using alternative, potentially more sensitive definitions of cardiotoxicity. Third, the predictors in our model are inherently limited to the variables selected by our feature selection process. Consequently, the model does not incorporate other clinically relevant factors, such as detailed family history or specific comorbidities. Therefore, it should be used as an adjunct to, rather than a replacement for, comprehensive clinical evaluation. Fourth, the retrospective nature of this study and the exclusion of a substantial number of patients (*n* = 345) due to incomplete data may introduce selection bias, potentially limiting the representativeness of our cohort. Future prospective studies with consecutive enrollment are needed to mitigate this limitation.

Our current model serves as a foundational step in this ongoing effort. By establishing a robust and interpretable ML framework with strong performance using readily available clinical data, we provide a platform upon which novel biomarkers can be incrementally integrated and their additive value quantified. The key limitations of our model in this context are its reliance on a predefined feature set and its development in a single-center cohort. To address these and improve our models in the future, we plan to perform the following actions: (i) conduct prospective, multicenter validation studies to enhance generalizability; (ii) actively collaborate with groups discovering novel biomarkers, systematically incorporating the most promising candidates (e.g., from proteomic or genomic studies) into our XGBoost framework to assess their incremental predictive power; and (iii) explore the inclusion of advanced echocardiographic parameters, such as myocardial strain, which were not available in the current dataset. This iterative process of validation and feature enhancement will be crucial for developing a truly comprehensive and clinically deployable risk stratification tool.

## 5 Conclusion

The XGBoost model demonstrated excellent performance in predicting CTRCD among breast cancer patients, with both strong discrimination and calibration. It can support early risk stratification and help guide personalized cardioprotective strategies, making it a promising tool for clinical application in cardio-oncology.

## Data Availability

The raw data supporting the conclusions of this article will be made available by the authors, without undue reservation.
